# Properties and Therapeutic Application of Bromelain: A Review

**DOI:** 10.1155/2012/976203

**Published:** 2012-12-10

**Authors:** Rajendra Pavan, Sapna Jain, Shraddha, Ajay Kumar

**Affiliations:** Department of Biotechnology, Institute of Biomedical Education and Research, Mangalayatan University, Aligarh 202145, India

## Abstract

Bromelain belongs to a group of protein digesting enzymes obtained commercially from the fruit or stem of pineapple. Fruit bromelain and stem bromelainare prepared differently and they contain different enzymatic composition. “Bromelain” refers usually to the “stem bromelain.” Bromelain is a mixture of different thiol endopeptidases and other components like phosphatase, glucosidase, peroxidase, cellulase, escharase, and several protease inhibitors. *In vitro* and *in vivo* studies demonstrate that bromelain exhibits various fibrinolytic, antiedematous, antithrombotic, and anti-inflammatory activities. Bromelain is considerably absorbable in the body without losing its proteolytic activity and without producing any major side effects. Bromelain accounts for many therapeutic benefits like the treatment of angina pectoris, bronchitis, sinusitis, surgical trauma, and thrombophlebitis, debridement of wounds, and enhanced absorption of drugs, particularly antibiotics. It also relieves osteoarthritis, diarrhea, and various cardiovascular disorders. Bromelain also possesses some anticancerous activities and promotes apoptotic cell death. This paper reviews the important properties and therapeutic applications of bromelain, along with the possible mode of action.

## 1. Introduction

Pineapple is the common name of *Ananas comosus *(*syns. A. sativus, Ananassa sativa, Bromelia ananas, B. comosa*). Pineapple is the leading edible member of the family Bromeliaceae, grown in several tropical and subtropical countries including Philippines, Thailand, Indonesia, Malaysia, Kenya, India, and China. It has been used as a medicinal plant in several native cultures [1] and these medicinal qualities of pineapple are attributed to bromelain (EC 3.4.22.32), which is a crude extract from pineapple that contains, among other compounds, various closely related proteinases, exhibiting various fibrinolytic, antiedematous, antithrombotic, and anti-inflammatory activities *in vitro *and* in vivo*. Bromelain has been chemically known since 1875 and is used as a phytomedical compound [2]. Bromelain concentration is high in pineapple stem, thus necessitating its extraction because, unlike the pineapple fruit which is normally used as food, the stem is a waste byproduct and thus inexpensive [3]. A wide range of therapeutic benefits have been claimed for bromelain, such as reversible inhibition of platelet aggregation, sinusitis, surgical traumas [4], thrombophlebitis, pyelonephriti angina pectoris, bronchitis [5], and enhanced absorption of drugs, particularly of antibiotics [6, 7]. Several studies have been carried out indicating that bromelain has useful phytomedical application. However, these results are yet to be amalgamated and critically compared so as to make out whether bromelain will gain wide acceptance as a phytomedical supplement [8]. Bromelain acts on fibrinogen giving products that are similar, at least in effect, to those formed by plasmin [9]. Experiment in mice showed that antacids such as sodium bicarbonate preserve the proteolytic activity of bromelain in the gastrointestinal tract [10]. Bromelain is considered as a food supplement and is freely available to the general public in health food stores and pharmacies in the USA and Europe [11]. Existing evidence indicates that bromelain can be a promising candidate for the development of future oral enzyme therapies for oncology patients [12]. Bromelain can be absorbed in human intestines without degradation and without losing its biological activity [12, 13].

## 2. Biochemical Properties

The crude aqueous extract from stem and fruit of pineapple is known as bromelain. It is a mixture of different thiol endopeptidases and other components like phosphatases, glucosidase, peroxidases, cellulases, glycoproteins, carbohydrates, and several protease inhibitors [14]. Stem bromelain (EC.3.4.22.32) is different from fruit bromelain (EC.3.4.22.33) [15]. The enzymatic activities of bromelain comprise a wide spectrum with pH range of 5.5 to 8.0 [16]. Different protein fractions were obtained by mean of various “biochemical techniques as sodium dodecyl sulphate polyacrylamide gel electrophoresis” (SDS-PAGE), isoelectric focusing (IEF), and multicathodal-PAGE [17, 18]. Nowadays, bromelain is prepared from cooled pineapple juice by centrifugation, ultrafiltration, and lyophilization. The process yields a yellowish powder, the enzyme activity of which is determined with different substrates such as casein (FIP unit), gelatin (gelatin digestion units), or chromogenic tripeptides [7, 17, 19, 20].

## 3. Absorption and Bioavailability

The body can absorb significant amount of bromelain; about 12 gm/day of bromelain can be consumed without any major side effects [13]. Bromelain is absorbed from the gastrointestinal tract in a functionally intact form; approximately 40% of labeled bromelain is absorbed from intestine in high molecular form [21]. In a study carried out by Castell et al. [13] bromelain was detected to retain its proteolytic activity in plasma and was also found linked with alpha 2-macroglobulin and alpha1-antichymotrypsin, the two antiproteinases of blood. In a recent study, it was demonstrated that 3.66 mg/mL of bromelain was stable in artificial stomach juice after 4 hrs of reaction and also 2.44 mg/mL of bromelain remained in artificial blood after 4 hrs of reaction [22].

## 4. Medicinal Uses

Clinical studies have shown that bromelain may help in the treatment of several disorders.

### 4.1. Effects of Bromelain on Cardiovascular and Circulation

Bromelain prevents or minimizes the severity of angina pectoris and transient ischemic attack (TIA). It is useful in the prevention and treatment of thrombophlebitis. It may also break down cholesterol plaques and exerts a potent fibrinolytic activity. A combination of bromelain and other nutrients protect against ischemia/reperfusion injury in skeletal muscle [23]. Cardiovascular diseases (CVDs) include disorders of the blood vessels and heart, coronary heart disease (heart attacks), cerebrovascular disease (stroke), raised blood pressure (hypertension), peripheral artery disease, rheumatic heart disease, heart failure, and congenital heart disease [24]. Stroke and heart disease are the main cause of death, about 65% of people with diabetes die from stroke or heart disease. Bromelain has been effective in the treatment of CVDs as it is an inhibitor of blood platelet aggregation, thus minimizing the risk of arterial thrombosis and embolism [25]. King et al. [26] reported that administration of medication use to control the symptoms of diabetes, hypertension, and hypercholesteromia increased by 121% from 1988–1994 to 2001–2006 (*P* < 0.05) and was greater for patients with fewer healthy lifestyle habits. Bromelain supplement could reduce any of risk factors that contribute to the development of cardiovascular disease. In a recent research, Bromelain was found to attenuate development of allergic airway disease (AAD), while altering CD4^+^ to CD8^+^T lymphocyte populations. From this reduction in AAD outcomes it was suggested that bromelain may have similar effects in the treatment of human asthma and hypersensitivity disorders [27]. In another study, carried out by Juhasz et al., Bromelain was proved to exhibit the ability of inducing cardioprotection against ischemia-reperfusion injury through Akt/Foxo pathway in rat myocardium [28].

### 4.2. Bromelain Relieves Osteoarthritis

Osteoarthritis is the most common form of arthritis in Western countries; in USA prevalence of osteoarthritis ranges from 3.2 to 33% dependent on the joint [29]. A combination of bromelain, trypsin, and rutin was compared to diclofenac in 103 patients with osteoarthritis of the knee. After six weeks, both treatments resulted in significant and similar reduction in the pain and inflammation [30]. Bromelain is a food supplement that may provide an alternative treatment to nonsteroidal anti-inflammatory drug (NSAIDs) [31]. It plays an important role in the pathogenesis of arthritis [32]. Bromelain has analgesic properties which are thought to be the result of its direct influence on pain mediators such as bradykinin [33, 34]. The earliest reported studies investigating bromelain were a series of case reports on 28 patients, with moderate or severe rheumatoid or osteoarthritis [35].

### 4.3. Effect of Bromelain on Immunogenicity

Bromelain has been recommended as an adjuvant therapeutic approach in the treatment of chronic inflammatory, malignant, and autoimmune diseases [36]. *In vitro* experiments have shown that Bromelain has the ability to modulate surface adhesion molecules on T cells, macrophages, and natural killer cells and also induce the secretion of IL-1*β*, IL-6, and tumour necrosis factor *α* (TNF*α*) by peripheral blood mononuclear cells (PBMCs) [37–43]. Bromelain can block the Raf-1/extracellular-regulated-kinase- (ERK-) 2 pathways by inhibiting the T cell signal transduction [44]. Treatment of cells with bromelain decreases the activation of CD4 (+) T cells and reduce the expression of CD25 [45]. Moreover, there is evidence that oral therapy with bromelain produces certain analgesic and anti-inflammatory effects in patients with rheumatoid arthritis, which is one of the most common autoimmune diseases [46].

### 4.4. Effect of Bromelain on Blood Coagulation and Fibrinolysis

Bromelain influences blood coagulation by increasing the serum fibrinolytic ability and by inhibiting the synthesis of fibrin, a protein involved in blood clotting [47]. In rats, the reduction of serum fibrinogen level by bromelain is dose dependent. At a higher concentration of bromelain, both prothrombin time (PT) and activated partial thromboplastin time (APTT) are markedly prolonged [48]. *In vitro* and *in vivo* studies have suggested that bromelain is an effective fibrinolytic agent as it stimulates the conversion of plasminogen to plasmin, resulting in increased fibrinolysis by degrading fibrin [49, 50].

### 4.5. Effects of Bromelain on Diarrhea

Evidence has suggested that bromelain counteracts some of the effects of certain intestinal pathogens like *Vibrio cholera* and *Escherichia coli, *whose enterotoxin causes diarrhoea in animals. Bromelain appears to exhibit this effect by interacting with intestinal secretory signaling pathways, including adenosine 3′ : 5′-cyclic monophosphatase, guanosine 3′ : 5′-cyclic monophosphatase, and calcium-dependent signaling cascades [51]. Other studies suggest a different mechanism of action. In *E. coli *infection, an active supplementation with bromelain leads to some antiadhesion effects which prevent the bacteria from attaching to specific glycoprotein receptors located on the intestinal mucosa by proteolytically modifying the receptor attachment sites [52, 53].

### 4.6. Effect of Bromelain on Cancer Cells

Recent studies have shown that bromelain has the capacity to modify key pathways that support malignancy. Presumably, the anticancerous activity of bromelain is due to its direct impact on cancer cells and their microenvironment, as well as on the modulation of immune, inflammatory, and haemostatic systems [12]. Most of the *in vitro *and *in vivo *studies on anticancer activity of bromelain are concentrated on mouse and human cells, both cancerous and normal, treated with bromelain preparations. In an experiment conducted by Beez et al chemically induced mouse skin papillomas were treated with bromelain and they observed that it reduced tumor formation, tumor volume and caused apoptotic cell death [54]. In one study related to bromelain treatment of gastric carcinoma Kato III cell lines, significant reduction of cell growth was observed [55] while in another study bromelain reduced the invasive capacity of glioblastoma cells and reduced *de novo* protein synthesis [56]. Bromelain is found to increase the expression of p53 and Bax in mouse skin, the well-known activators of apoptosis [54]. Bromelain also decreases the activity of cell survival regulators such as Akt and Erk, thus promoting apoptotic cell death in tumours. Different studies have demonstrated the role of NF-*κ*B, Cox-2, and PGE2 as promoters of cancer progression. Evidence shows that the signaling and overexpression of NF-*κ*B plays an important part in many types of cancers [57, 58]. Cox-2, a multiple target gene of NF-*κ*B, facilitates the conversion of arachidonic acid into PGE2 and thus promotes tumour angiogenesis and progression [59, 60]. It is considered that inhibiting NF-*κ*B, Cox-2, and PGE2 activity has potential as a treatment of cancer. Bromelain was found to downregulate NF-*κ*B and Cox-2 expression in mouse papillomas [54] and in models of skin tumourigenesis [61]. Bromelain was also shown to inhibit bacterial endotoxin (LPS)-induced NF-*κ*B activity as well as the expression of PGE2 and Cox-2 in human monocytic leukemia and murine microglial cell lines [62, 63]. Bromelain markedly has *in vivo *antitumoural activity for the following cell lines: P-388 leukemia, sarcoma (S-37), Ehrlich ascetic tumour, Lewis lung carcinoma, and ADC-755 mammary adenocarcinoma. In these studies, intraperitoneal administration of bromelain after 24 hours of tumour cell inoculation resulted in tumour regression [54].

### 4.7. Role of Bromelain in Surgery

Administration of bromelain before a surgery can reduce the average number of days for complete disappearance of pain and postsurgery inflammation [64, 65]. Trials indicate that bromelain might be effective in reducing swelling, bruising, and pain in women having episiotomy [66]. Nowadays, bromelain is used for treating acute inflammation and sports injuries [31].

### 4.8. Role of Bromelain in Debridement Burns

The removal of damaged tissue from wounds or second/third degree burns is termed as debridement. Bromelain applied as a cream (35% bromelain in a lipid base) can be beneficial for debridement of necrotic tissue and acceleration of healing. Bromelain contains escharase which is responsible for this effect. Escharase is nonproteolytic and has no hydrolytic enzyme activity against normal protein substrate or various glycosaminoglycan substrates. Its activity varies greatly with different preparations [67]. In two different enzymatic debridement studies carried out in porcine model, using different bromelain-based agents, namely, Debriding Gel Dressing (DGD) and Debrase Gel Dressing showed rapid removal of the necrotic layer of the dermis with preservation of the unburned tissues [68, 69]. In another study on Chinese landrace pigs, enzymatic debridement using topical bromelain in incised wound tracks accelerated the recovery of blood perfusion, pO_2_ in wound tissue, controlled the expression of TNF-*α*, and raised the expression of TGT-*β* [70]. Enzymatic debridement using bromelain is better than surgical debridement as surgical incision is painful, nonselective and exposes the patients to the risk of repeated anaesthesia and significant bleeding [71–74].

### 4.9. Toxicity of Bromelain

According to Taussig et al. [75] bromelain has very low toxicity with an LD_50_ (lethal doses) greater than 10 g/kg in mice, rates, and rabbits. Toxicity tests on dogs, with increasing level of bromelain up to 750 mg/kg administered daily, showed no toxic effects after six months. Dosages of 1500 mg/kg per day when administered to rats showed no carcinogenic or teratogenic effects and did not provoke any alteration in food intake, histology of heart, growth, spleen, kidney, or hematological parameters [76]. Eckert et al. [41] after giving bromelain (3000 FIP unit/day) to human over a period of ten days found no significant changes in blood coagulation parameters.

## 5. Conclusion

Bromelain has a wide range of therapeutic benefits, but the mode of its action is not properly understood. It is proved that bromelain is well absorbed in body after oral administration and it has no major side effects, even after prolonged use. All the evidences reviewed in this paper suggest that bromelain can be used as an effective health supplement to prevent cancer, diabetes, and various cardiovascular diseases in the long run.

## 6. Future Trends and Perspectives

Bromelain can be a promising candidate for the development of oral enzyme therapies for oncology patients. It is clear from this paper that bromelain is a multiaction enzyme; however, more research is required to understand the proper mechanism of action of bromelain so that the multiaction activities of bromelain can be harnessed efficiently.
